# Visual Stability and the Motion Aftereffect: A Psychophysical Study Revealing Spatial Updating

**DOI:** 10.1371/journal.pone.0016265

**Published:** 2011-01-26

**Authors:** Ulrich Biber, Uwe J. Ilg

**Affiliations:** Hertie-Institute for Clinical Brain Research, Department of Cognitive Neurology, University of Tübingen, Tübingen, Germany; Istituto di Neuroscienze, Italy

## Abstract

Eye movements create an ever-changing image of the world on the retina. In particular, frequent saccades call for a compensatory mechanism to transform the changing visual information into a stable percept. To this end, the brain presumably uses internal copies of motor commands. Electrophysiological recordings of visual neurons in the primate lateral intraparietal cortex, the frontal eye fields, and the superior colliculus suggest that the receptive fields (RFs) of special neurons shift towards their post-saccadic positions before the onset of a saccade. However, the perceptual consequences of these shifts remain controversial. We wanted to test in humans whether a remapping of motion adaptation occurs in visual perception.

The motion aftereffect (MAE) occurs after viewing of a moving stimulus as an apparent movement to the opposite direction. We designed a saccade paradigm suitable for revealing pre-saccadic remapping of the MAE. Indeed, a transfer of motion adaptation from pre-saccadic to post-saccadic position could be observed when subjects prepared saccades. In the remapping condition, the strength of the MAE was comparable to the effect measured in a control condition (33±7% vs. 27±4%). Contrary, after a saccade or without saccade planning, the MAE was weak or absent when adaptation and test stimulus were located at different retinal locations, i.e. the effect was clearly retinotopic.

Regarding visual cognition, our study reveals for the first time predictive remapping of the MAE but no spatiotopic transfer across saccades. Since the cortical sites involved in motion adaptation in primates are most likely the primary visual cortex and the middle temporal area (MT/V5) corresponding to human MT, our results suggest that pre-saccadic remapping extends to these areas, which have been associated with strict retinotopy and therefore with classical RF organization. The pre-saccadic transfer of visual features demonstrated here may be a crucial determinant for a stable percept despite saccades.

## Introduction

When we move our eyes, the resulting retinal slip cannot be distinguished from global movement of the surrounding environment at the retinal input level. However, the primate visual system can compensate for rapid eye movements also known as saccades, which occur at a frequency of 3/s during our waking hours [1]. Consequently, we do *not* perceive shifts of the environment during the execution of saccades. As has been proposed early by von Helmholtz [2], this spatial stability may be maintained by subtracting an internal reference signal from the retinal motion signal. The reafference principle [3] and the corollary discharge theory [4] explain this in the following way: a reafferent signal and an efference copy or corollary discharge signal are used to create a difference signal called exafference or comparator output, which is conveyed to higher centers of the brain allowing to filter changes out of perception, which are caused by own eye movements. The reafferent signal arises from sensory activation within the effector, in this example the retina. The efference copy is equivalent to the internal reference signal proposed by von Helmholtz. In this case it is a copy of the eye movement command. Following this theory, voluntary eye movements create an exafference of zero, given a stationary environment and head position. In principle, this could explain why we perceive a stable visual environment across different fixations. An impressive experiment to test this theory is to immobilize the eyes by a paralytic drug and have the subject try to move his eyes. Kornmüller [5] did this in a self-experiment and reported that each intended eye movement was accompanied by a shift or displacement of the environment (Umweltverlagerung) in the same direction the intended eye movement was aiming. Similar experiments were carried out by Stevens et al. [6] and Matin et al. [7] involving partial or total paralysis of the eye muscles and also complete neuromuscular paralysis. According to the reafference principle, abolishing retinal reafferent information would leave efference copies to solely determine the exafference. Seemingly, it is the efference copy that creates the perception of displacement of the environment when a subject with paralyzed eye muscles *tries* to move his eyes but actually cannot do so. These experiments serve as strong evidence for the reafference principle and more specifically suggest the physiological existence of efference copies. Regarding saccades it has been suggested by Sommer and Wurtz that in monkeys a pathway originating from the superior colliculus (SC), passing through mediodorsal thalamus, reaching cortex at the frontal eye field, carries the efference copy signal [8]. In their experiments a special task that necessitates internal monitoring of saccades called the double-step task [9], [10] was used. In this task, subjects are instructed to perform two sequential memory-guided saccades (termed 1^st^ and 2^nd^ saccade hereafter) to previously cued locations, i.e. there is no retinal feedback about the current eye position. Sommer and Wurtz tested their hypothesis by temporarily lesioning the mediodorsal thalamus while having monkeys perform the described double-step task [11]. In order to make 2^nd^ saccades correctly, monkeys had to factor the 1^st^ saccade into generation of the 2^nd^ saccade, i.e. the 2^nd^ saccade depended on efference copies. Indeed, monkeys showed systematic errors in 2^nd^ saccade endpoints but not in 1^st^ saccade endpoints following muscimol injections into mediodorsal thalamus. Thus, it was concluded that efference copies are used to coordinate sequential saccades. In humans, Gaymard and colleagues described two patients with central thalamic lesions suffering from a deficit comparable to the monkeys' impairment [12]. In their study, patients were asked to perform memory-guided saccades but with an intervening eye displacement either caused by visually-guided saccades, a smooth tracking eye movement or a whole body movement. The patients showed markedly impaired saccade accuracy compared to a simple memory-guided saccade paradigm and compared to a healthy control group. Also in humans, Heide et al. measured the ability of 35 patients with unilateral focal cortical lesions to perform a double-step saccade paradigm [13]. The range of lesions included posterior parietal cortex, prefrontal cortex and the assumed locations of the human frontal and supplementary eye fields. Patients with lesions in posterior parietal cortex showed the highest frequency of erroneous 2^nd^ saccades. The authors concluded that posterior parietal cortex is crucial for spatial constancy across saccades. The case of patient R.W. with bilateral extrastriate cortical lesions in areas 18, 19 and possibly 37 suffering from false perception of motion has been described by Haarmeier and colleagues [14]. While performing smooth tracking eye movements, R.W. perceived retinal slip induced by background motion as though it were motion in extrapersonal space. Only if the background was stabilized on the retina, R.W. perceived it as stationary. Indeed, the perception of very small movements of the stationary surround may also occur in healthy humans during smooth tracking eye movements. This motion is commonly referred to as Filehne illusion [15]. There is also evidence that impaired efference copies are part of the pathology of schizophrenia [16], [17], [18] and may underlie different characteristic sensory and motor deficits caused by brain lesions [19], [20], [21], [22], [23]. The case of R.W. demonstrates that the ability to perceive a stationary world despite own eye movements can be selectively impaired. To date, the mechanisms that maintain spatial constancy are still being investigated [24]. An important principle that emerged from recent discoveries is the dynamic receptive field (RF). Traditionally, it has been assumed that visual RFs are constant with respect to spatial and stimulus selectivity, i.e. the so called classical RF exhibits a static mapping. However, it is now clear that RFs are much more dynamic and may access information from outside the classical RF under certain conditions, e.g. when an eye movement is planned. Single-unit recordings in the lateral intraparietal area (LIP) of monkeys performed by Duhamel and Goldberg [25] suggest that most of these neurons' RFs shift from pre- to post-saccadic positions prior to the execution of visually driven saccades. They described two distinctive properties of these neurons in more detail. Firstly, they shift their RF from pre- to post-saccadic or future position shortly before the saccade begins (16 of 36 cells), and secondly, they seem to carry a memory trace of targets flashed shortly (50 ms) within the future RF (22 of 23 cells). The term future RF again refers to the extension or jump of the RF towards the post-saccadic position shortly before the execution of the saccade. The updating or remapping is hypothesized to work in the following way: upon appearance of a stimulus, neurons with RFs covering the stimulus' position increase their activity. When a saccade is planned, shortly before its execution, these neurons are thought to transfer their activity to neurons whose RFs will encompass the stimulus location after the saccade [26]. It remained unclear, however, whether efference copies drive the remapping process. Later experiments by Sommer and Wurtz [27] investigating properties of neurons in the saccadic subregion of the cortical frontal eye field (FEFsac) of macaque monkeys demonstrated that these neurons show impaired visual processing, i.e. defective remapping, when the mediodorsal thalamus, supposedly the relay station for the efference copy signals, was temporarily inactivated. Eventually, remapping has been observed on a single-unit level in a number of brain areas including LIP [25], [28], FEF [29], [30], the intermediate layers of the SC [31] and in extrastriate visual cortex in areas V3 and V3a [32]. Altogether, there seems to be circumstantial evidence that remapping is an important factor for maintenance of spatial constancy [33], [34]. Until this day, remapping has been demonstrated within the visual domain on a single-unit level in monkeys, as described above, and also on a population level using functional magnetic resonance imaging (fMRI) in humans. Merriam et al. described voxels showing remapped activity in extrastriate visual areas while performing saccadic eye movements [35]. However, it has not been shown yet experimentally whether remapping directly influences visual motion perception per se or, alternatively, is merely important to the oculomotor system ensuring accuratesequences of saccades. In our study, we address this question and ask whether motion adaptation is suitable for revealing pre-saccadic remapping in visual cognition. To this end, we employed an adaptation paradigm involving the motion aftereffect (MAE). This is an exceptionally stable illusion that occurs after viewing of coherent motion, in the way that static images appear to be moving in the opposite direction of adaptation [36], [37]. For example, an adapter stimulus of random dots moving leftwards is presented for a couple of seconds while a subject fixates in the center of this stimulus. After this motion adaptation, static dots at the same location, serving as test stimulus, will appear to be moving rightwards. Importantly, this seems not to be the case if the test stimulus is presented at a different retinal location, i.e. the reference frame of the MAE occurs to be retinotopic [38], [39], [40]. Moreover, there are only few studies suggesting that motion information is combined across glances [41], [42], [43], [44], [45], [46]. In the critical condition of our paradigm the test stimulus was flashed within the future RFs of neurons, which were supposedly adapted beforehand. Thus, the strength of the illusion or size of the MAE was a measure for the strength of remapping taking place. In this way, we were asking whether pre-saccadic remapping has an impact on human visual perception. We were also trying to deduce whether this process has an impact on brain areas involved in motion adaptation. We found coherent psychophysical evidence for pre-saccadic remapping of the motion aftereffect in human subjects. This suggests that the remapping mechanism is a crucial component for conveying visual constancy.

## Results

**Figure 1:**
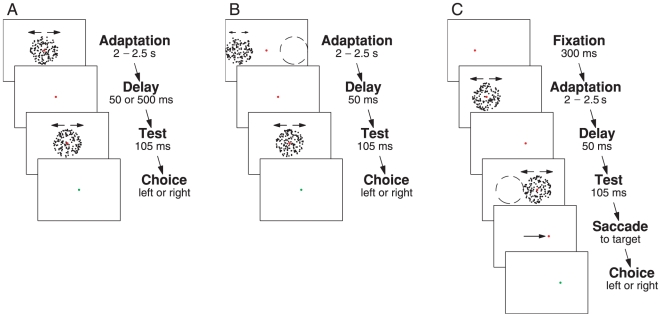
Experimental Paradigm. In each experiment, subjects were instructed to fixate a small red square in the center of an adapter stimulus, which consisted of a random dot kinematogram (RDK) shown within a stationary circular aperture for a random time period lasting between 2 and 2.5 s. The adapter stimulus' dots moved either left- or rightwards at 3°/s. The test stimulus was flashed briefly and was either static or moving slowly left- or rightwards at 0.6 or 1.2°/s. Subjects reported moving direction of the test stimulus by a keypress upon appearance of a small green square. **A** Baseline and Storage of the MAE: Both fixation target and adapter stimulus were centered on the screen. After a delay of 50 or 500 ms the test stimulus was shown centrally as well. **B** Retinal Specificity or Phantom MAE: The adapter stimulus was shown 14° left or right (dashed circle) from the fixation target while subjects fixated in the center, where the test stimulus was shown with a delay of 50 ms. **C** Pre-saccadic Remapping of the MAE: The fixation target, which was shown 7° left or right from the center, was presented for 300 ms before adaptation started. Subjects were instructed to make a saccade upon appearance of a red square at the beginning of the delay period (third panel). The saccade target was always located on the opposite side of the screen relative to the adapter stimulus. Conditions for rightward saccades are depicted. Location and timing of the test stimulus were controlled separately. It appeared either 50 or 500 ms after offset of the adapter stimulus, and it was shown either centered around the position of the original fixation target (dashed circle) or centered around the position of the saccade target. The illustrations are drawn to scale (width: 42.5° height: 32°) and fixation and saccade targets were red. Dashed circles and arrows were not part of the display.

To answer the question whether pre-saccadic remapping of the locus of the MAE occurs, we designed three specific experiments, which are depicted in Figure 1. In the first experiment the baseline MAE and storage MAE size was addressed (Figure 1A). In the second experiment, we measured retinal specificity or the so called ‘phantom’ MAE (Figure 1B). In the third experiment, we addressed remapping of the MAE (Figure 1C).

### Experiment 1: Baseline and Storage of the Motion Aftereffect

The purpose of this experiment was to measure the size of the ‘standard’ MAE in our subjects in conditions comparable to the pre-saccadic remapping experiment (Experiment 3). We quantified the subjective magnitude of the MAE induced by two different delay durations between adapter and test stimulus (50 and 500 ms). The experimental procedure is depicted in Figure 1A. We controlled eye movements to ensure eye fixation and analysed responses made by key presses of seven human subjects in a two alternatives forced choice paradigm (2AFC). About 3% of all 4200 trials were excluded from the analysis due to breaks of fixation caused by eye blinks or saccades.

**Figure 2:**
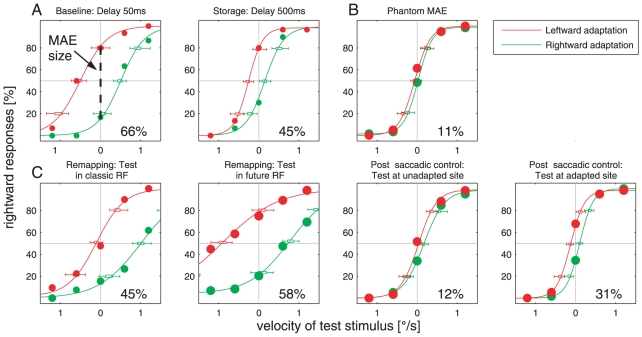
Psychometric Functions of a Representative Subject (S.F.) for all three Experiments. In each panel, the percentage of the subject's rightward choices is plotted against the test stimulus' velocity. The diameter of the data points reflects the number of measurements in each condition. In principle, we measured 30 trials in each condition. But note that in B and C data were collapsed from mirror-inverted conditions, yielding 60 measurements in each condition. **A** The baseline and storage experiment is shown with delays of 50 and 500 ms. Red and green data points represent responses following left- and rightward adaptation, respectively. Logistic functions and error bars from bootstrap runs are colored accordingly (see Experimental procedures for more details). MAE size estimates were obtained from the difference between the percentage of rightward responses, following left- and rightward adaptation, upon presentation of static test stimuli, marked by the intercept of the logistic functions with the y-axis (left panel). In the left panel, the test stimulus was shown 50 ms after presentation of the adapter stimulus (baseline condition). In the right panel, the test stimulus was shown with a delay of 500 ms (storage condition). **B** Retinal Specificity or Phantom MAE: Adaptation was either in the left or right periphery, whereas the test stimulus was shown centrally after a delay of 50 ms. Data from both adaptation loci did not differ significantly and were pooled for clarity. **C** Pre-saccadic Remapping of the MAE: Data from rightward and leftward saccade trials representing mirror-inverted conditions were collapsed for clarity. Only the four principle conditions are shown, in which adapter and test stimulus were either on the same or opposite sides, and the delay of the test stimulus was either 50 or 500 ms. The first two panels represent remapping conditions (delay 50 ms), where the test stimulus was either shown at the fixation target (leftmost panel) or at the saccade target (middle left panel). The last two panels depict post-saccadic control conditions (delay 500 ms), where the test stimulus was shown either at the original fixation target (middle right panel) or at the already fixated saccade target (rightmost panel). Labeling of B and C as explained in A.

**Figure 3:**
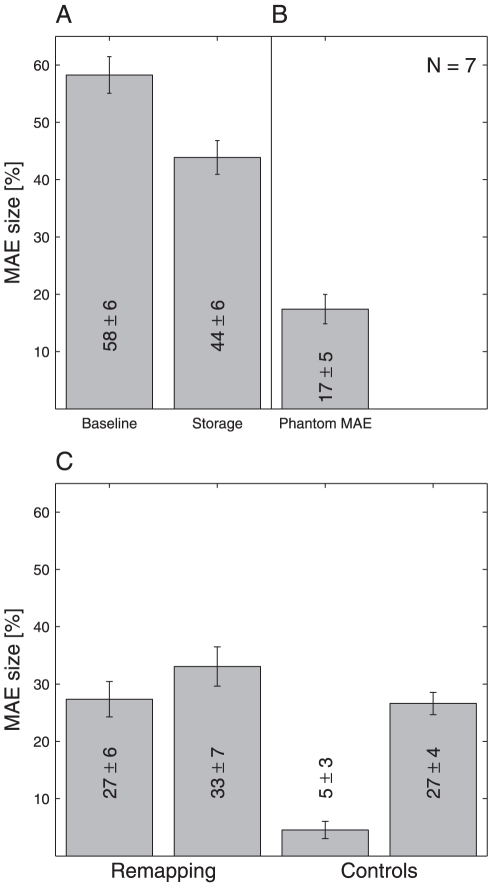
Size of the Motion Aftereffect in All Three Experiments. Bars show means and error bars represent SEM across subjects (N = 7). **A** MAE size of both the baseline and storage experiment are shown with delays 50 and 500 ms, respectively. **B** Size of the Phantom MAE: Data from both adapter locations (right and left periphery) were collapsed. **C** MAE size in the pre-saccadic remapping experiment: Data from rightward and leftward saccade trials representing mirror-inverted conditions were collapsed. Bars are shown in the same order as psychometric functions in Figure 2C. In the first remapping condition (leftmost bar) the test stimulus was shown at the fixation target shortly before the saccade, i.e. in the classical RFs of presumably motion adapted neurons. In the second remapping condition (second leftmost bar) the test stimulus was shown at the saccade target but shortly before the saccade, i.e. following the remapping hypothesis in the future RFs of presumably motion adapted neurons. In the first control condition (second rightmost bar) the test stimulus was shown after the saccade at an unadapted peripheral site. In the second control condition (rightmost bar) the test stimulus was shown after the saccade but at the adapted central retinal site.

Figure 2 shows psychometric functions for a representative subject (S.F.) for all three experiments. In Figure 2A, the baseline condition (delay 50 ms) is shown in the left panel, and the storage condition (delay 500 ms) appears in the right panel. In each panel, percentage of rightwards responses is plotted versus the velocity of the test stimulus. Negative values correspond to leftward motion and positive values to rightward motion. Note that there is also a specific condition in which the test stimulus was stationary. Data points, psychometric functions and error bars for leftward adaptation are plotted in red and those for rightward adaptation are plotted in green. The intersection points of the best fitting logistic functions of both directions of adaptation with the y-axis served as a basis for the MAE size estimate. The difference between these intersection points for leftward and rightward adaptation was used to quantify the magnitude of the MAE, observed in a single subject. The MAE size for this subject is given in the lower right corner of each panel. As shown in Figure 3A, the mean MAE size across all seven subjects in the baseline condition (delay 50 ms) was 58±6% SEM, whereas the mean MAE size for the storage condition (delay 500 ms) was 44±6% SEM. Concisely, increasing the delay duration from 50 to 500 ms reduced the MAE to 75% of its original size. A two-factorial ANOVA yielded highly significant effects of both the factors experimental condition (baseline vs. storage, P = 0.0038; F = 20.9) and the random effects factor subject (P = 0.0025; F = 14.3).

### Experiment 2: Retinal Specificity of the Motion Aftereffect

Not only the MAE but most visual aftereffects are retinotopic, i.e. the effect is only present when the part of the retina that was adapted also senses the test stimulus. This has been demonstrated e.g. for the MAE induced by linear motion [47] or the spiral MAE [48]. However, the MAE shows interocular transfer [47] and partial transfer to adjacent locations, termed ‘remote’ or ‘phantom’ MAE [49], [50]. The purpose of this experiment was to obtain precise information about the size of the phantom MAE in our setting. This was necessary because a strong phantom MAE would have been impossible to discern from remapping. We controlled eye movements and examined responses from seven human subjects after presentation of a peripheral adapter stimulus and a central test stimulus as depicted in Figure 1B. About 8% of all 4200 trials were excluded from the analysis due to breaks of fixation. Psychometric functions of one representative subject (S.F.) are shown in Figure 2B. Since there was no significant difference regarding MAE size between the adapter loci (14 degrees left or right from the fixation point), data from both conditions were pooled. The large overlap of data points from leftward- and rightward adaptation trials (red and green) indicate that there was no significant difference between both functions, and therefore phantom MAE size was very small in this subject (11%). Mean size of the phantom MAE across all subjects was 17±5% SEM, as is shown in Figure 3B. The phantom MAE was significantly smaller than both the baseline (t-test, P = 0.0003) and the storage condition (t-test, P = 0.005) of the first experiment. Also, it was significantly larger than zero (t-test against zero, P = 0.0144). However, considering single subjects, 4 out of 7 pairs of psychometric functions for leftward and rightward adaptation were not differing significantly (Monte Carlo test, P>0.05). Summing up, the phantom MAE was weakly manifest but was not a stringent phenomenon across all subjects.

### Experiment 3: Pre-saccadic Remapping of the Motion Aftereffect

**Figure 4:**
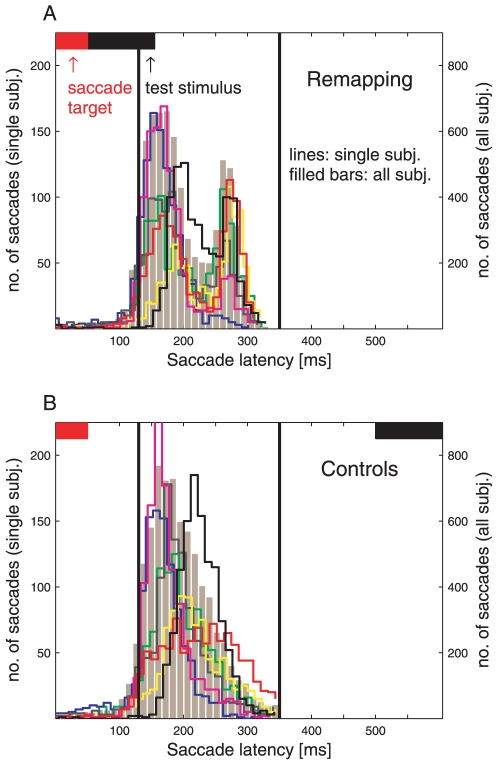
Saccade Latencies in the Remapping Experiment. Number of saccades in each subject (scale on left y-axis) and mean of all subjects (scale on right y-axis) are plotted against saccade latency. Total saccade count is shown as ochre bar histogram. For clarity, histograms of single subjects are depicted by colored stair functions. All histograms are made up of 35 bins, equivalent to a bin size of approximately 11 ms. The horizontal red and black bars depict times of saccade target and test stimulus presentation. The two black vertical lines enclose the interval of saccade latencies chosen for analysis. **A** Saccade latencies for trials with a delay period of 50 ms (remapping conditions). **B** Saccade latencies for trials with a delay period of 500 ms (control conditions).

In our main experiment, we searched for evidence for pre-saccadic remapping of visual space as revealed by the motion aftereffect. We inspected eye movements and responses from seven human subjects in a saccade paradigm designed to reveal possible pre-saccadic remapping of the locus of the MAE. About 20% of all 16800 trials were excluded from the analysis mainly due to artefacts caused by eye blinks and inappropriate saccade latency (see Figure 4 and Experimental procedures for further details). We also controlled for saccade parameters (Figure S1, showing saccade duration and peak velocity). Approximately 1900 trials recorded from each of seven subjects were analysed. Representative results of one subject are shown in Figure 2C, and means are shown in Figure 3C. The first two panels of Figure 2C and Figure 3C represent remapping conditions. In the first case, when adapter and test stimulus are on one side and the delay between the two is 50 ms, a saccade is being prepared at the very moment the test stimulus is shown, whilst the subject's eyes are still stationary. In this condition MAE size decreased to 27±6% SEM (Figure 3C), which was smaller than MAE size in the baseline condition of the first experiment (t-test, P = 0.0047), but was not significantly different from phantom MAE size (t-test, P = 0.2385). In the second case, when adapter and test stimulus are on opposite sides and the delay between the two is 50 ms, the saccade is being prepared at the same time the test stimulus is shown. However, here the test stimulus is presented at future RF positions of motion adapted neurons. In this condition, which is depicted in Figure 1C, we found the strongest of motion aftereffects in Experiment 3: 33±7% SEM, less than the baseline MAE from Experiment 1 (t-test, P = 0.0200) but significantly larger than phantom MAE size from Experiment 2 (t-test, P<0.0460). The third and fourth panels show post-saccadic control conditions. In the third panel, adapter and test stimulus are on the same side but with a delay of 500 ms between the two. The test stimulus was shown after the saccade and at a non-adapted position in retinal coordinates. This negative control condition can be viewed as a ‘storage phantom motion aftereffect’ with a delay of 500 ms. We found the weakest aftereffect in this condition: 5±3% SEM. It was not different from zero (t-test, P = 0.1816) and thus significantly smaller than any other aftereffect measured in all three experiments. In the fourth panel, adapter and test stimulus were on opposite sides and were presented with a delay of 500 ms. The test stimulus was shown after the saccade and at the same retinal coordinates where the adapter was displayed. This positive control condition is closest to the storage condition of the first experiment with the difference of an intervening saccade between presentation of adapter and test stimulus. Mean effect size in this condition was 27±4% SEM, which is smaller than the storage condition of the first experiment (t-test, P = 0.0309), but not different from both remapping conditions (t-tests, comparison with first remapping condition: P = 0.9200; comparison with second remapping condition, P = 0.4287). In a nutshell, we found the strongest of motion aftereffects in the crucial remapping condition.

## Discussion

In this study, we report the presence of a MAE in a visual remapping paradigm. Our motivation was to investigate remapping processes in the context of visual constancy. As we summarized in Figure 3, our data shows a profound MAE in the crucial remapping condition that was significantly larger than the phantom MAE, which served as a control. We conclude, firstly, that pre-saccadic remapping has an impact on human visual perception, evident as a modulation of the MAE at the current and future RF. This extends the remapping theory by showing that spatial updating is not limited to static features, but is also present for motion features. Secondly, we hypothesize that low-level visual areas should exhibit remapping properties. Our data supports the first conclusion, whereas the second is speculative. The discussion especially aims to clarify this speculative conclusion. Therefore, we discuss the neuronal substrates that are involved in motion adaptation and remapping. Candidate neuronal substrates involved in motion adaptation are early cortical visual areas such as V1, V2, V3, V3A, V4 and also areas MT/V5 and MST, because they contain directionally selective cells. Directionality is an indicator for the involvement of a neuron in motion processing. The proportion of directionally selective neurons, in macaque monkey cortex, varies across the mentioned areas from roughly 13% in area V4 [51], 12–15% in area V3 [52], [53], about one quarter to one third in V1 [54], [55] and unclear proportions in V2 and V3A. By far the largest proportion of directionally selective cells of roughly 90% can be found in area MT of several species of both New and Old World monkeys [52], [56], [57], [58], [59], [60], [61]. Neurons in area MST are also directionally selective, but are optimally driven by more complex motions such as expansion and contraction [62], and thus should not be compared directly to cells in the other areas mentioned. The firing rate of directionally selective cells drops following motion adaptation in their preferred direction, which has been demonstrated in single-units in cat V1 [63],[64], monkey V1 [65], owl monkey MT [66] and in macaque MT [65], [67], [68], [69]. On a population level, using an fMRI adaptation paradigm in monkeys, Tolias et al. [70] have shown that areas V1, V2/V3, V3A, V4 and MT are directionally tuned with the strongest selectivity in MT and V4. The activation of V4, however, may have been artificial according to the authors. Moreover, activity of MT cells shows correlation to the perception of motion direction (for reviews see [71], [72]). For example, Newsome and colleagues trained macaques in a 2AFC direction discrimination task to measure motion coherency thresholds in terms of both psychophysical performance and neuronal responses of MT cells simultaneously [73]. Psychophysical and neural performance matched well both with respect to slopes and sensitivity of neurometric and psychometric functions. Furthermore, it has been shown that motion thresholds are selectively elevated following MT lesioning [74], and cortical microstimulation in area MT introduces a bias in perceptual judgments towards the motion direction encoded by the stimulated neurons [75]. Consequently, cells in MT have been presumed to underlie the MAE [66]. These numerous findings from animal testing are supplemented by few studies on directional sensitivity in humans. Using an fMRI adaptation paradigm, Huk et al. [76] provided evidence for directional selectivity which was strongest in MT+ and weaker in areas V1 and V2. Note that the distinction between human MT and human MST seems to be difficult in fMRI. That is why the MT/MST areas are frequently referred to as MT+ or motion complex. Another fMRI study in humans [77] showed that at least area MT and almost certainly area MST are motion sensitive in a direction-selective manner. Another fMRI study by Tootell et al., which directly tried to map the neuronal substrate of the MAE, identified human MT as the most responsive area during experience of the aftereffect [78]. Moreover, time courses of the psychophysical-MAE and the fMRI-MAE were very similar. It has been argued by Huk et al. [76], that it was merely attention that created the effect observed by Tootell and colleagues. However, they found in their own study that imbalances in MT+ responses underlie the MAE. In the broader sense, one should also consider that motion adaptation does not occur on a single cortical stage, but may take place on multiple levels. For instance, it has been argued that static and dynamic MAEs can be attributed to adaptation at different cortical sites due to differences in perception regarding for example optimal adaptation speed [79] or bandwidth tuning of adaptation motion [80].

Considering the findings regarding directional selectivity and motion adaptation, we discuss how this might be related to the pre-saccadic transfer of the MAE and spatial updating. Remapping was first described in visuo-motor area LIP of macaques. With its powerful saccade-related activity and its reciprocal connections to other saccade centers, this area is also known as parietal eye field [81], [82]. It is noteworthy that LIP is also closely linked to spatial attention, which seems to be locked to the position of a saccade target shortly before a saccade [83]. Regarding area MT, the most plausible candidate for perception of the MAE, no remapping properties have been described so far, but another form of RF plasticity has been demonstrated in this area by Kohn and Movshon [84]: motion adaptation in one part of the RF did not induce a decreased response to a test stimulus in a different part of the RF. This suggests that MT adaptation is inherited from V1 cells. Otherwise, one would expect that adaptation in one part of a RF affects the whole RF. Furthermore, there is evidence that spatial attention causes dynamic shifts and shrinking of RFs around the attended stimulus in area MT [85]. At least, this demonstrates that RFs in area MT are not static but highly plastic. Regarding area V1, another candidate for perception of the MAE, it has been shown that there is a fast post-saccadic restoration of attentional modulation, which occurs 47 ms earlier than if a new stimulus is presented [86]. This can also be interpreted as a correlate of trans-saccadic integration.

In humans, again there is little evidence, but presumably remapped activity has been found in striate and extrastriate cortex using fMRI [35]. The investigators suggest that the strength of remapping is roughly monotonically increasing with position in the visual hierarchy, i.e. remapped responses are strongest in V3A and hV4 and smallest in V1 and V2. Cortical areas outside the occipital lobe were not investigated in this study. Another electrophysiological correlate of remapping in humans has been identified employing scalp-recorded EEG [87]. Subjects made saccades that caused a visual stimulus either to remain within a visual hemifield, or to cross the vertical meridian. In the latter case, pre-saccadic potentials showed increased bilaterality. However, the source of the remapping responses could not be assessed in this study.

A remaining question is how the remapping signal reaches the neurons adapted in our paradigm, which may be located in early visual areas and/or the human motion complex. It was speculated earlier that remapping observed in LIP is driven by signals from the saccade region of the frontal eye fields (FEFsac) [88]. This is supported by anatomical studies showing reciprocal connections between these two areas [89], [90], as well as by a functional study using a delayed saccade task [91]. Although, in this study, the functional connectivity is described to be biased towards the visual modality, saccade-related responses were also present. Monosynaptical connections between FEF/FEFsac and MT/MST have also been identified by tracer injections [92], [93], [94]. Only recently, the involvement of MT/MST in processing or receiving saccade-related oculomotor information has been discovered in monkeys performing memory saccades in complete darkness [95]. However, there is no direct evidence for a functional relationship between FEF and MT/MST. An alternative route of the remapping signal from LIP to MT/MST is at least supported by anatomical evidence for reciprocal connections between these areas [82], [90]. Finally, an influence from the SC to areas MT and MST should be considered. The inferior pulvinar of the monkey is known to be both a recipient of SC input as well as a source of projections to area MT [96], [97], [98]. However, lesioning SC has little impact on properties of MT cells regarding directional selectivity, orientation tuning, RF size, or binocularity [99]. In combination with our data, this suggests that the remapping signal arises from the SC, passes through the human analogue of FEF, or through LIP, altering neuronal properties of V1 and/or MT+ cells, and creates the observed MAE.

Regarding the “remapped MAE”, one might ask why it was weaker than the baseline MAE (57%). The most parsimonious explanation should be that not all neurons responsible for perception of the MAE show remapped activity. At the same time, this could explain why the MAE was not eliminated when subjects prepared to make a saccade away from the test stimulus (47%). Favouring this explanation is the fact that the combined MAEs from both remapping conditions add up to be as large as the baseline MAE. Moreover, we can exclude that a lack of retinal specificity is responsible for our findings, since the observed phantom MAE was significantly weaker than the remapped MAE (53%). In a recent psychophysical study by Melcher [100], addressing the tilt aftereffect (TAE), it was also an important prerequisite to show that this illusion is retinotopic. Subjects were adapted to tilted static gratings and afterwards judged purely vertical test gratings as tilted towards the opposite direction. It was demonstrated that this TAE occurred at the future gaze position shortly before a saccade and, in contrast, was significantly reduced when the test was presented at the position of the adapter. The transfer of the TAE to the future gaze position is consistent with the transfer of the MAE we observed. However, the reduction of the TAE when the test was shown at the adapter position at first glance seems to be more pronounced than the reduction of the MAE that we observed in this condition. We propose that this is due to differences in the test stimuli used in Melcher's and our study: duration of 50 ms vs. 105 ms, static vs. moving, and differences in timing with respect to the saccade onset. In Melcher's study trials were sorted based on the onset of the test stimulus relative to saccade onset and he found the strongest decrease near the saccade onset but a somewhat weaker decrease when the test stimulus was shown right after the saccade cue, as was the case in our experiment. To control for retinotopy, Melcher used adapters located 4 or 7 degrees above or below the central fixation point, as well as test gratings around that fixation point. Alternatively, the adapter was shown at the fixation point, and the test was shown ten degrees in the periphery. Only in case of adapters located 4 degrees above or below the fixation point a TAE roughly 30% of the original TAE size was observed, which is comparable to the size of our phantom MAE compared to the baseline MAE (29%).

There is more evidence that the MAE is not entirely retinotopic. Meng, Mazzoni & Qian [101] showed transfer of the MAE to non-adapted locations using expansion motion but no transfer for translational motion. Regarding linear motion there is clear evidence that the MAE is strongest at the adapted location [49], but partial transfer to adjacent regions has been reported as well [49], [50]. Essentially, we can verify that partial transfer to adjacent locations occurs. However, this was not a consistent phenomenon across all individuals. These variable responses give reason to speculate, that for example attentional differences may have elicited the phantom MAE, which we observed in some of our subjects.

Storage of the MAE is supposed to be best, i.e. surviving long delays, when subjects close their eyes between adaptation and test stimulus [47]. It has been demonstrated that the nature of the intervening storage pattern is relatively unimportant, as long as it is not identical to the adaptation stimulus [102]. Moreover, storage is more complete in the case of dynamic compared to static test patterns [103]. The latter we used in our experiment in first approximation. The decay of the aftereffect (76% of baseline) was expected in our delay condition (500 ms) and seems to accurately reflect the storage property of the MAE. Since the time constant of the decay critically depends on the presentation duration of the adapter, which was shorter than in most studies addressing the MAE, we cannot compare our findings with decay times from other studies. However, we could observe that the decay of the MAE was much stronger (47% of baseline) after the same delay of 500 ms, when an intervening saccade was introduced. Therefore, one might speculate that execution of saccades speeds up the decay of the MAE.

In conclusion, the findings of our study imply that remapping processes, as revealed by shifting of the locus of the MAE, extend to low level visual areas. In monkeys, this hypothesis could be tested experimentally in area V1 or MT/MST using the same approaches that were applied in area LIP and FEF, which means flashing stimuli in the future RFs of the recorded neurons. In humans, it could be tested using fMRI and a saccade paradigm, revealing remapped activity. Such experiments could verify our results and change the present view on primate primary visual cortex and the motion complex. Traditionally, it has been assumed that neuronal properties of these so called low-level visual areas represent relatively simple transformations of the retinal input. However, more recent and also our findings cast severe doubt on this notion. It appears that dynamic RFs and remapping processes are much more common and widespread phenomena in visual processing than proposed to date. These pre-saccadic alterations may be responsible for smooth trans-saccadic perception. Furthermore, the remapping of motion information should be important for the survival of all kinds of animals, which move the eyes to accurately track movements of both predator and prey.

## Materials and Methods

### Ethics Statement

All participants gave oral informed consent prior to taking part in the experiments. From each participant, it was documented that he or she gave oral consent before the experiment started. Since the study involved exclusively non-invasive perceptual measurements, no written consent was given or approval from the ethics committee was required.

### Subjects, Apparatus and Eye Movement Recordings

Seven healthy human subjects (2 female and 5 male) aged between 21 and 33 years (mean age 24.3±4.1 SD) participated in each of three experiments described below. The experiments were performed with the understanding and consent of each subject. All subjects had normal or corrected to normal vision. All experiments were performed in a darkened room. Stimuli were presented on a 19 in. CRT-Screen (Iiyama Vision Master Pro 454 HM903DT B driven by a NVIDIA GeForce 6600GT video card) at a viewing distance of 44 cm, resulting in maximal display area of 47° horizontally and 35° vertically. All spatial linear dimensions and velocities will be given in degrees or arcmins and degrees per second, computed at the tangent point at the center of the monitor. Spatial resolution was ∼34 px/deg in both horizontal and vertical directions, corresponding to 1600×1200 pixels total screen resolution. The vertical refresh rate was 104.5 Hz. All stimuli were custom-made and written in C including the Simple DirectMedia Layer (SDL) library. Horizontal and vertical eye positions were recorded from the right and left eye, respectively, using an infrared eye tracker (IRIS Skalar) with a spatial resolution of 0.2°. The analog signals were low-pass filtered (corner frequency: 100 Hz) and digitized at a temporal resolution of 1 kHz.

### Experiment 1: Baseline and Storage of the Motion Aftereffect

As depicted in Figure 1A, subjects viewed an adapter stimulus consisting of a random-dot kinematogram (RDK) with a diameter of 14° on a black background in the center of the screen. Parameters of the RDK are described in more detail below. The dots were moving coherently either left- or rightwards at a velocity of 3°/s, while subjects were fixating a small red square (edge length 6.5 arcmin) in the center of the stimulus. The adapter stimulus was shown for a random duration lasting between 2 and 2.5 s. After a delay of either 50 ms or 500 ms, during which fixation was maintained, a test stimulus, also consisting of a RDK with a central red square, was shown for 105 ms. Next, subjects judged the moving direction of the test stimulus in a two alternatives forced choice (2AFC) manner using key presses, ‘1’ for left and ‘0’ for right. We informed our subjects that *only* horizontal moving directions occurred and that they should make a decision even if the test stimulus was perceived as stationary. Each of 20 conditions (2 directions of adaptation ×2 delay durations ×5 velocities of the test stimulus) was presented 30 times in a pseudo-randomized order in three blocks of 200 trials each, totaling 600 trials per subject.

### Experiment 2: Retinal Specificity of the Motion Aftereffect

Subjects viewed an adapter stimulus as described in Experiment 1 but positioned peripherally with its center either 14° right or left from the central fixation target, as shown in Figure 1B. Note that there was no spatial overlap between adapter and test stimulus. The test stimulus was shown after 50 ms for a duration of 105 ms in the center of the screen and subjects judged the moving direction of the test stimulus as in Experiment 1. All 20 conditions (2 adapter positions ×2 directions of adaptation ×5 velocities of the test stimulus) were displayed 30 times in pseudo-randomized order in three blocks of 200 trials each, totaling 600 trials per subject.

### Experiment 3: Presaccadic Remapping of the Motion Aftereffect

Initially, the adapter position was cued by a red square for 300 ms whilst subjects were fixating or returning to the fixation target (see Figure 1C). Subsequently the adapter stimulus was presented, positioned peripherally with its center either 7° right or left from the middle of the screen. Next, a saccade target, a red square with an edge length of 6.5 arcmin, was shown on the other side of the screen, i.e. 14° left or right from the fixation target depending on initial stimulus position. This saccade target was followed by the presentation of the test stimulus either with a delay of 50 or 500 ms, i.e. the test stimulus was presented either before of after the saccade. Furthermore, the test stimulus could either be shown at the initial fixation position or at the position of the saccade target. Finally, subjects judged the moving direction of the test stimulus (Fig. 1C). All 80 conditions (2 adapter positions ×2 directions of adaptation ×2 delay durations ×2 test stimulus positions ×5 velocities of the test stimulus) were presented 30 times in pseudo-randomized order in ten blocks of 240 trials each, totaling 2400 trials per subject.

### Properties of the Random-Dot Kinematograms

Both adapter and test stimulus RDKs were presented within a circular area with a diameter of 14°. There was no physical border surrounding adapter or test stimulus. The test stimulus was either stationary or moving slowly left- or rightwards at 0.6°/s or 1.2°/s. The dots were white squares with an edge length of 3.2 arcmin corresponding to 2 by 2 pixels. These squares created the impression of filled circles, due to their very small size. Dot density was 4 dots/deg^2^. Luminance of the adapter stimulus' dots was 6 cd/m^2^ and luminance of the test stimulus' dots was 92 cd/m^2^. Luminance of the background was below the luminance meter's threshold. Each dot was initiated with a random lifetime between 10 and 402 ms (1 to 42 frames). As soon as the lifetime of a single dot ceased, it re-entered at a random position within the stimulus area with a lifetime of 402 ms. However, lifetime of the test stimulus' dots was fixed to 105 ms (11 frames), because pilot experiments indicated that accuracy of discrimination was very poor for test stimuli with random lifetimes, presumably due to additional flicker introduced by random lifetimes. As soon as a dot would have vanished from the circular area a y-axis mirroring transformation was applied to it and consequently the dot reappeared on the other side of the aperture.

### Psychometric Functions and Goodness of Fit

All data processing was performed using Matlab. Psychometric functions were fitted using the psignifit toolbox version 2.5.6 for Matlab (see http://bootstrap-software.org/psignifit/), which implements the maximum-likelihood method described by Wichmann and Hill [104]. Goodness of fit and comparison of psychometric functions were also assessed using the psignifit toolbox. The estimation of goodness of fit yielded positive results for almost all psychometric curves of the seven subjects (100% in the baseline/storage experiment; 100% in the phantom MAE experiment; 96.4% in the remapping experiment). The comparison of psychometric curves for left- and rightward adaptation indicated significant differences between the functions (7/7 subjects in the baseline/storage experiment; 3/7 subjects in the phantom MAE experiment; in the remapping experiment: 5/7 in the first remapping condition, 6/7 in the second remapping condition, 0/7 in the negative control and 7/7 in the positive control condition).

### Manual Reaction Times

The time interval between onset of the go-signal (last panel in each part of Figure 1) and a subject's keypress was defined as manual reaction time (MRT). Trials with a MRT above 1500 ms were discarded from the analysis. We performed a two-factorial ANOVA on MRTs with the factors experiment and subject. Results of this analysis are described in Figure S2. There was no significant difference between the experiments but significant differences between the subjects regarding MRTs. However, there was a tendency towards longer reaction times in the remapping experiment.

### Analysis of Eye Movement Recordings

The entire data analysis of eye position profiles was performed on the basis of single trials. Horizontal as well as vertical eye velocity and acceleration were calculated by differentiation of the eye position data. Eye position profiles were low-pass filtered at 40 Hz, eye velocity profiles at 10 Hz (Butterworth, first order). Polar velocity and acceleration were calculated from combined horizontal and vertical profiles. Saccade detection was optimized for large saccades with amplitudes around 15°. More precisely, saccade onset was detected when polar acceleration exceeded 2500°/s^2^. Temporal offset of the first saccade with respect to appearance of the saccade target is referred to as saccade latency. In the first and second experiment, we excluded trials in which fixation was broken by saccades occurring during the last 500 ms of fixation, the delay period or during presentation of the test stimulus. In the third experiment, trials with too short (<130 ms) or too long (>350 ms) saccade latency or no saccade at all within this time period were excluded from the analysis (see Figure 4).

## Supporting Information

Figure S1 **Saccade Parameters in the Remapping Experiment.** **A** Saccade duration of single subjects (1-7) and mean for all saccades. Error bars depict standard deviations. Subjects 2, 3, 4, 6 and 7 show no significantly different saccade durations (ANOVA F = 116, P<0.001 & post-hoc Scheffé-test). 3 out of 7 subjects (1, 3 and 6) had a significant directional bias (left- vs. rightward) for saccade duration (t-test). **B** Saccade peak velocity. Labeling as in A. Comparing subject 1 with 2 as well as subject 2 with 5 yielded no significant difference in saccade peak velocities, whereas all other comparisons were significant. A significant directional bias regarding saccade peak velocity was present in 5 out of 7 subjects (2, 3, 5, 6 and 7).
(EPS)

Figure S2 **Manual Reaction Times in all three Experiments.** In each panel, histograms (bin size 50 ms) from all but the removed trials (originally 4200 in A and B, 16800 in C) of all 7 subjects are shown. Vertical black lines denote median manual reaction times. Trials with reaction times above 1500 ms are not shown and were discarded from the psychophysical analysis. **A** Baseline/Storage experiment. Trials from the baseline condition (50 ms delay) and the storage condition (500 ms delay) are pooled. **B** Retinal Specificity or Phantom MAE. Trials from both adapter loci (left and right periphery) are pooled. **C** Pre-saccadic remapping. Trials from all different conditions (different delay times, adapter stimulus positions and test stimulus positions) were pooled. A two-factorial ANOVA yielded no significant effect for the factor experiment (F = 1.13; P = 0.38) and a highly significant random effects factor subject (F = 11.35; P<0.001). However, there was no significant interaction between the two factors (F = 0.78; P = 0.66). A post-hoc Scheffé test revealed significant inter-subject differences between all subjects but two.
(EPS)
